# Personalized Antibiotic Therapy for the Critically Ill: Implementation Strategies and Effects on Clinical Outcome of Piperacillin Therapeutic Drug Monitoring—A Descriptive Retrospective Analysis

**DOI:** 10.3390/antibiotics10121452

**Published:** 2021-11-26

**Authors:** Schrader Nikolas, Riese Thorsten, Kurlbaum Max, Meybohm Patrick, Kredel Markus, Surat Güzin, Scherf-Clavel Oliver, Strate Alexander, Pospiech Andreas, Hoppe Kerstin

**Affiliations:** 1 Department of Anesthesiology, Intensive Care, Emergency and Pain Medicine, University Hospital Wuerzburg, Oberdürrbacher Str. 6, 97080 Wuerzburg, Germany; Riese_T@ukw.de (R.T.); Meybohm_P@ukw.de (M.P.); Markus.Kredel@kliniken-ostalb.de (K.M.); Hoppe_K1@ukw.de (H.K.); 2 Department of Internal Medicine I, Division of Endocrinology/Diabetology, University Hospital Wuerzburg, Oberdürrbacher Str. 6, 97080 Wuerzburg, Germany; Kurlbaum_M@ukw.de; 3 Central Laboratory, University Hospital Wuerzburg, Oberdürrbacher Str. 6, 97080 Wuerzburg, Germany; Strate_A@ukw.de; 4 Unit for Infection Control and Antimicrobial Stewardship, University Hospital Wuerzburg, 97080 Wuerzburg, Germany; Surat_G@ukw.de; 5 Institute for Pharmacy and Food Chemistry, University of Wuerzburg, Am Hubland, 97074 Wuerzburg, Germany; Scherf-Clavel_O@ukw.de; 6 Pharmacy, University Hospital Wuerzburg, Innere Aumühlstr. 3, 97076 Wuerzburg, Germany; Pospiech_A@ukw.de

**Keywords:** therapeutic drug monitoring, piperacillin/tazobactam, personalized antimicrobial therapy, antimicrobial stewardship

## Abstract

Therapeutic drug monitoring (TDM) is increasingly relevant for an individualized antibiotic therapy and subsequently a necessary tool to reduce multidrug-resistant pathogens, especially in light of diminishing antimicrobial capabilities. Critical illness is associated with profound pharmacokinetic and pharmacodynamic alterations, which challenge dose finding and the application of particularly hydrophilic drugs such as β-lactam antibiotics. *Methods:* Implementation strategy, potential benefit, and practicability of the developed standard operating procedures were retrospectively analyzed from January to December 2020. Furthermore, the efficacy of the proposed dosing target of piperacillin in critically ill patients was evaluated. *Results*: In total, 160 patients received piperacillin/tazobactam therapy and were subsequently included in the study. Of them, 114 patients received piperacillin/tazobactam by continuous infusion and had at least one measurement of piperacillin serum level according to the standard operating procedure. In total, 271 measurements were performed with an average level of 79.0 ± 46.0 mg/L. Seventy-one piperacillin levels exceeded 100 mg/L and six levels were lower than 22.5 mg/L. The high-level and the low-level group differed significantly in infection laboratory parameters (CRP (mg/dL) 20.18 ± 11.71 vs. 5.75 ± 5.33) and renal function [glomerular filtration rate (mL/min/1.75 m^2^) 40.85 ± 26.74 vs. 120.50 ± 70.48]. *Conclusions:* Piperacillin levels are unpredictable in critically ill patients. TDM during piperacillin/tazobactam therapy is highly recommended for all patients. Although our implementation strategy was effective, further strategies implemented into the daily clinical workflow might support the health care staff and increase the clinicians’ alertness.

## 1. Introduction

Sepsis is defined as a life-threatening organ dysfunction due to a dysregulated host response to bacteria or their components [1]. An estimated incidence of 48.9 million sepsis cases were recorded in 2017 worldwide, resulting in 11 million sepsis related deaths, thereby contributing to a global overall lethality of 20% [2].

Antimicrobial therapy is an essential key issue in the management of patients with bacterial infections, sepsis, and septic shock. Inappropriate empirical antimicrobial therapy results in significantly increased morbidity and mortality [3]. Every delay in the application of an adequate antimicrobial therapy causes an increased mortality [4]. Several mechanisms including the release of vasodilative mediators and cytokines as well as the activation of immune cells affect nearly all aspects of endothelial cell function, subsequently resulting in impaired vasoregulation, barrier function, inflammation, and hemostasis [5]. The resulting capillary leakage combined with the necessary aggressive volume therapy might increase the volume of distribution significantly, finally risking sub-therapeutic antibiotic drug levels. Additionally, several further organ dysfunctions impair the pharmacokinetics and pharmacodynamics of drugs during sepsis. The hyperdynamic phase during sepsis is usually associated with an increased cardiac output and renal clearance of particularly hydrophilic antibiotics, while acute renal failure increases the distribution volume. Moreover, antibiotic drug levels might be influenced by hypoalbuminemia or impaired hepatobiliary metabolism, both of which are regularly associated with sepsis. Numerous studies have reported that antibiotic plasma levels are highly variable and unpredictable in critically ill patients. Plasma levels of a significant part of critically ill patients do not achieve the pharmacokinetic/dynamic targets, subsequently increasing the likelihood of therapeutic failures and development of bacterial resistance or achieving toxic serum concentrations [6,7,8].

To overcome these uncertainties and in view of diminishing antimicrobial capabilities, therapeutic drug monitoring (TDM) is a necessary tool to optimize antimicrobial treatment and to stop the continuing emergence of antimicrobial resistance [9].

β-Lactams such as piperacillin/tazobactam (PIP/TAZ) and carbapenems are frequently applied drugs for empirical antimicrobial therapy to treat sepsis and septic shock. Piperacillin/tazobatam is a broad-spectrum antibiotic with high in vitro activity against aerobic and anaerobic Gram-positive and Gram-negative pathogens including *Pseudomonas aeruinosa* [10]. Generally, the relevant issue for adequate antibacterial activity of β-lactams is the period with free drug concentration exceeding the minimum inhibitory concentration (fT_Mic_). A minimum period of at least 40% of fT_>MIC_ was reported to be clinically efficient [11], however, extended periods might be required for optimized bactericidal effects in critically ill patients [12,13]. In particular, β-lactams are highly hydrophilic drugs, which at least in part explain the high variability in pharmacokinetics and pharmacodynamics reported, especially in critically ill patients.

## 2. Results

### 2.1. Study Population

With a total of 742 patients treated from January to December 2020, 160 patients received PIP/TAZ at least once during an intensive care unit stay. The mean age of patients was 61 ± 16 years with 52 female and 108 male patients. The average height was 172.7 ± 10.0 cm, the average weight was 87.4 ± 22.6 kg, and the resulting average body mass index was 29.2 ± 7.3 kg/m^2^. Among all included patients, 122 (76.3%) patients suffered from respiratory insufficiency, 54 (33.8%) from acute respiratory distress syndrome, 114 (71.3%) from circulatory insufficiency, 12 (7.5%) from acute kidney injury, and six (3.8%) were polytrauma patients. Among these, 65 (40.6%) received dialysis and 38 (23.8%) died. During the observation period, 114 (70%) received TDM of piperacillin.

### 2.2. Measurement of Piperacillin Levels

**Figure 1:**
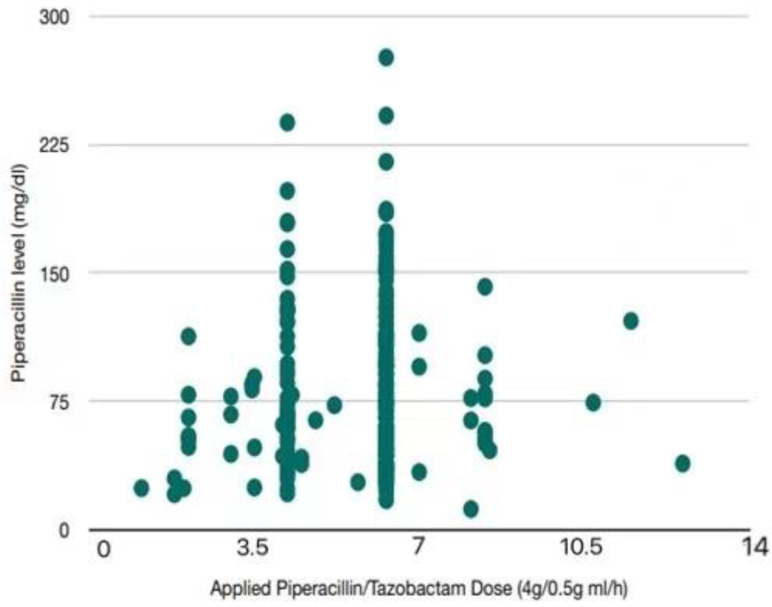
Illustration of applied dosage periods of piperacillin/tazobactam and the corresponding measured piperacillin blood level concentrations (*n* = 271). Piperacillin levels mg/L (y-axis) were shown in dependence of applied doses (x-axis). One dose was determined as 4 g PIP/TAZ independent of application duration.

Depending on treatment duration, patients received different amounts of TDM (49 patients one time; 23 patients two times; 16 patients three times; 14 patient four times; six patients five times; two patients six times; two patients seven times; and two patients eight times). In total, 271 piperacillin levels were measured, with a mean level of 79.0 ± 46.0 mg/L [minimum 12.1 mg/L and maximum 275.0 mg/L). In 71 measurements (26.2%), piperacillin levels exceeded 100 mg/L and in six measurements (2.2%), the measured doses were lower than 22.5 mg/L (one sample lower than 16 mg/L). In 84 measurements (31.0%), the measured piperacillin levels resulted in dose adaptions. In 30 measurements (11.1%), dose adaptions were performed due to initial piperacillin levels above 100 mg/L (Figure 1).

Piperacillin levels correlated with creatinine (ρ = 0.499), glomerular filtration rate (ρ = 0.521), number of platelets (ρ = 0.292), and procalcitonin (ρ = 0.500).

Clinical laboratory differences between piperacillin levels in, above, or below the recommended range are shown in Table 1. Significant differences between the low and the target level group were detected in markers of inflammation [CRP (mg/dL) target level: 16.9 ± 12.0; low level: 5.8 ± 5.3; IL-6 (pg/mL) target level: 501 ± 2866; low level: 49 ± 69] and the mean age [target level (y): 62 ± 14; low level group 47 ± 10]. Significant differences between the high and the target level groups were detected in severity of disease [CRP (mg/dL) target level: 16.9 ± 12.0; high level: 20.2 ± 11.7; PCT (pg/mL) target level: 4.8 ± 11.4; high level: 18.8 ± 36.6] and renal function [glomerular filtration rate (mL/min/1.75 m^2^) target level: 75.42 ± 51.75; high level: 40.85 ± 26.74; creatinine (mg/mL) target level: 1.41 ± 0.84; high level: 2.19 ± 0.97].

### 2.3. Outcomes

In total, 160 patients received PIP/TAZ treatment during their stay in the ICU. A total of 114 patients received TDM of piperacillin (TDM-group) and 46 received non-TDM therapy with PIP/TAZ (non-TDM group). The duration of PIP/TAZ application in the ICU was shorter than 48 h in 23 patients and therefore these patients were excluded from further analysis. Thirty-three patients were excluded from mortality analysis due to additional COVID infection. Mortality tended to be higher in the non-TDM group (TDM group: *n* = 12 [11%]; non-TDM group: *n* = 6 [24%].

### 2.4. Implementation of TDM

Although TDM has been implemented as a new standard method since the beginning of 2020, 23 patients did not receive TDM during PIP/TAZ therapy while staying for at least more than 72 h at the ICU. Length of ICU stay and the mean PIP/TAZ application period differed between both groups (ICU stay (days) TDM group: 15.9 ± 13.3; non-TDM group: 4.2 ± 8.7); application period (days) TDM group: 8.4 ± 4.7; non-TDM group: 4.4 ± 2.0).

## 3. Discussion

Therapeutic drug monitoring of antimicrobial pharmacy is increasingly relevant to ensure optimized treatment for selected patients, particularly critically ill patients with high and unpredictable variances in pharmacokinetics and dynamics, but also to avoid the development of augmented bacterial resistance. Personalized medicine is currently of pronounced interest and the progress in the development of biosensor technologies as so called point of care testing might also be promising [14,15,16,17]. However, at present, the most frequently applied technique to conduct TDM is based on immunological assays, available in commercial kits. Although these tests are rapid and cheap, standardization and calibration are challenging [16]. Therefore, the optimal diagnostic choice currently seems to be chromatography combined with mass spectrometry (LC-MS), which is mostly based on locally developed standards [16]. Although the application of LC-MS requires a sophisticated infrastructure including trained personnel and extensive apparatuses, the number of hospitals incorporating LC-MS in routine analysis is increasing [18]. This might be explained due to decreased costs of LC-MS equipment compared to earlier eras, but also as a consequence of improved sensitivity and speed [18,19]. The application of this technique enables fast turnaround times, which in turn benefits patient care. Although the measurement per se of about 30 min is relatively quick, usual turnaround times range between 18–24 h. To address this issue, we implemented an “in-house” TDM measurement with sample testing from 8 a.m. to 5 p.m., which subsequently guarantees turnaround times within 24 h, at least three times a week.

Continuous or prolonged (to at least 40–50% of the dosing interval) application of β-lactam antibiotics were reported to cause increased (e.g., 1–4x MIC) and extended (e.g., 100% fT_MIC_) blood antimicrobial drug levels compared to intermitted infusion in critically ill patients [20,21,22]. In order to avoid sub-therapeutic antimicrobial levels during a continuous or prolonged dose regime, daily TDM is highly recommended in several guidelines [23,24]. Based on this knowledge, we implemented a continuous PIP/TAZ application with blood level monitoring. Following this application regime, we measured levels between 22.5 mg/L and 100 mg/L in 71% of cases, while 2.2% of the measurements were below the ECOFF of *Pseudomonas aeruginosa*. Although this study reports mere observational retrospective data, the results may suggest that a continuous dose application might be superior to an intermitted bolus application. Previous data reported a target blood level of 100% fT_>MIC_ in 63% of cases during intermittent bolus therapy with β-lactam antibiotics [25]. Similarly, Chiriac et al. reported that the combined application of TDM and continuous dose application caused a concentration of piperacillin of 100% fT_4xMIC_ in 49% of the cases and 100% fT_>MIC_ in 99% of the cases, within the first 48 h after onset of treatment [26].

Otherwise, 26.2% of the measurements exceeded 100 mg/L by application of our regime, which were mainly after the application of the fourth and six doses. Dose induced toxicity of piperacillin was suggested to induce neurological deterioration or acute kidney injury. Both clinical symptoms are frequently associated with sepsis and septic shock and cannot be attributed to antimicrobial therapy alone, and were not specifically the focus of this study. The generally accepted breakpoints are currently under debate. Some authors have proposed a target of level of 100% fT_4-10xMIC_, based on the hypothesis that the therapeutic drug levels are decreased in the target compartment compared to the blood concentrations [27,28]. However, several recent studies have reported that a target attainment of 100% fT_4×MIC_ caused no additional benefit for the patients and might even increase mortality [29,30,31]. Based on these data, a target level of 80 mg/L seems reasonable for empiric antimicrobial therapy, but once the causative pathogens are identified, switching antimicrobial treatment to a tailored regime is the preferred approach.

The results of this study suggest that the blood levels of piperacillin highly depend on sepsis related impairment of organ dysfunction reflected by increased inflammation parameters and renal function.

Data analysis of the TDM group revealed that the sub-therapeutic patient collective showed significantly lower mean age, lower inflammatory parameters, and higher glomerular filtration rate—suggesting that the initial hyperdynamic septic phase is associated with increased clearance of piperacillin [26,30]. An early randomized study compared continuous versus discontinuous administration of PIP/TAZ and revealed that all patients treated with continuous infusion of 13.5 g/24 h had a free piperacillin concentration far above the highest MIC observed (i.e., 100% fT_>MIC_), while patients treated with discontinuous infusion of 3.375 g/6 h had free piperacillin concentration above the MIC for only 50% of the dosing interval (i.e., 50% fT_>MIC_) [24,32]. In contrast, population pharmacokinetic modeling data suggest the necessity of dose adaption in dependence of application mode, and higher daily doses might potentially be required to achieve target attainment during continuous infusion of PIP/TAZ [33]. However, whether or not piperacillin elimination is saturable at therapeutic plasma concentration is currently a matter of debate and needs to be clarified in further studies [34,35,36,37]. Nevertheless, some of the patients with sub-therapeutic levels might potentially be under-dosed. Therefore, we adapted our algorithm and added an additional dose regime for patients with augmented renal clearance [37,38] (Figure 1).

Otherwise, piperacillin levels exceeding the potential toxic level of 100 mg/L were measured in blood from patients with impaired renal function (median GFR 40.9 ± 26.8 mL/min/1.73 m^2^), which is in line with several previous studies reporting a strong correlation between impaired renal function and increased piperacillin blood levels [26,39,40]. However, the adaption of loading dose in dependence of renal function is currently under discussion. In line with the hypothesis that β-lactam antibiotics elimination is independent of renal function during the early infection period and to prevent sub-therapeutic blood levels, some authors recommend a starting fixed dose independent of renal function [29,41]. Several recent data pursuant to our results demonstrated that up to 30% of patients exceeded serum concentration of >100 mg/L within the first 48 h after beginning piperacillin treatment by continuous application [26]. Therefore, a strict control of piperacillin serum levels is highly favorable in this patient cohort to avoid levels exceeding 100 mg/L. In this respect, randomized studies are necessary to evaluate a “best practice” concept.

**Figure 2:**
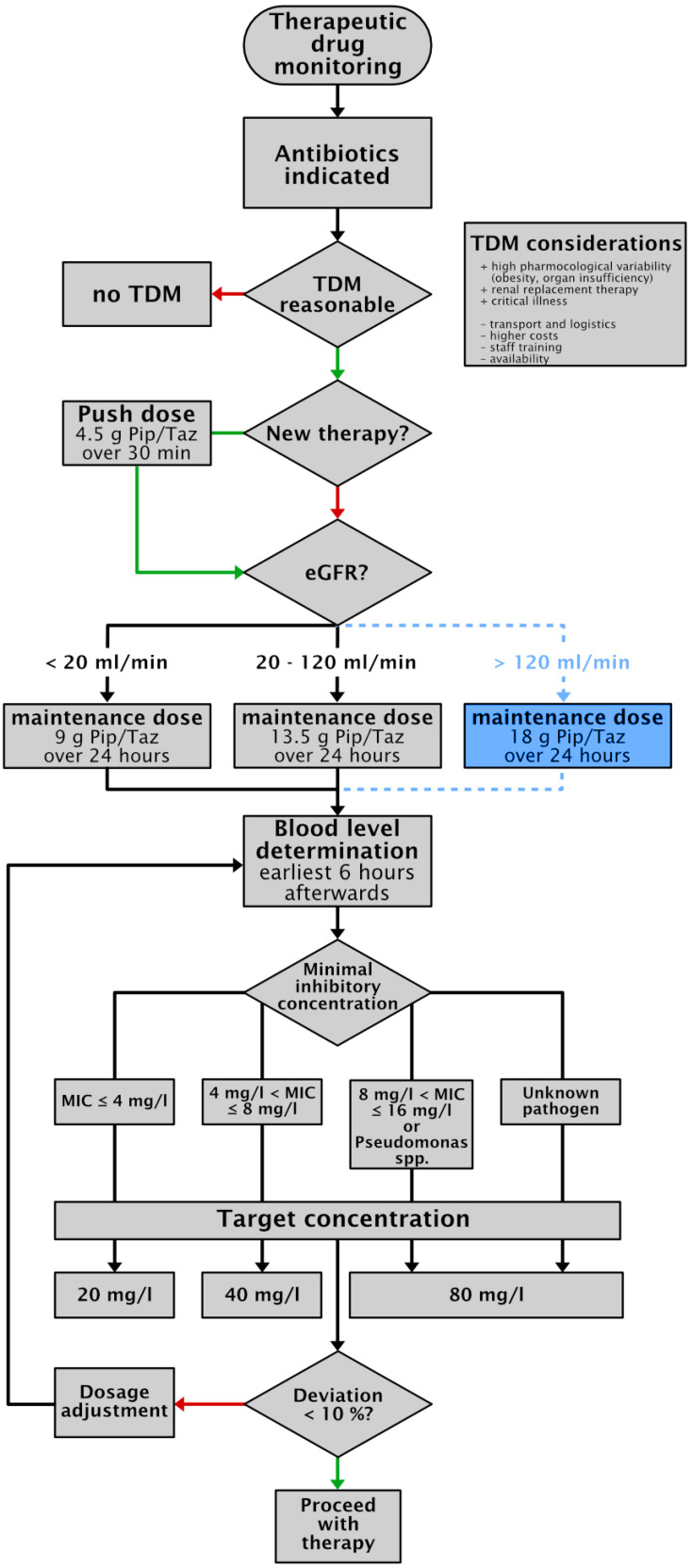
Implemented algorithm for the application of piperacillin/tazobactam and therapeutic drug monitoring of piperacillin at the University Hospital Wuerzburg. Based on the data of this study and the recent literature, we added a potential additional dose regime for critically ill patients with augmented renal clearance, which has to be approved by TDM level evaluation (blue box) [34,37,38].

**Figure 3:**
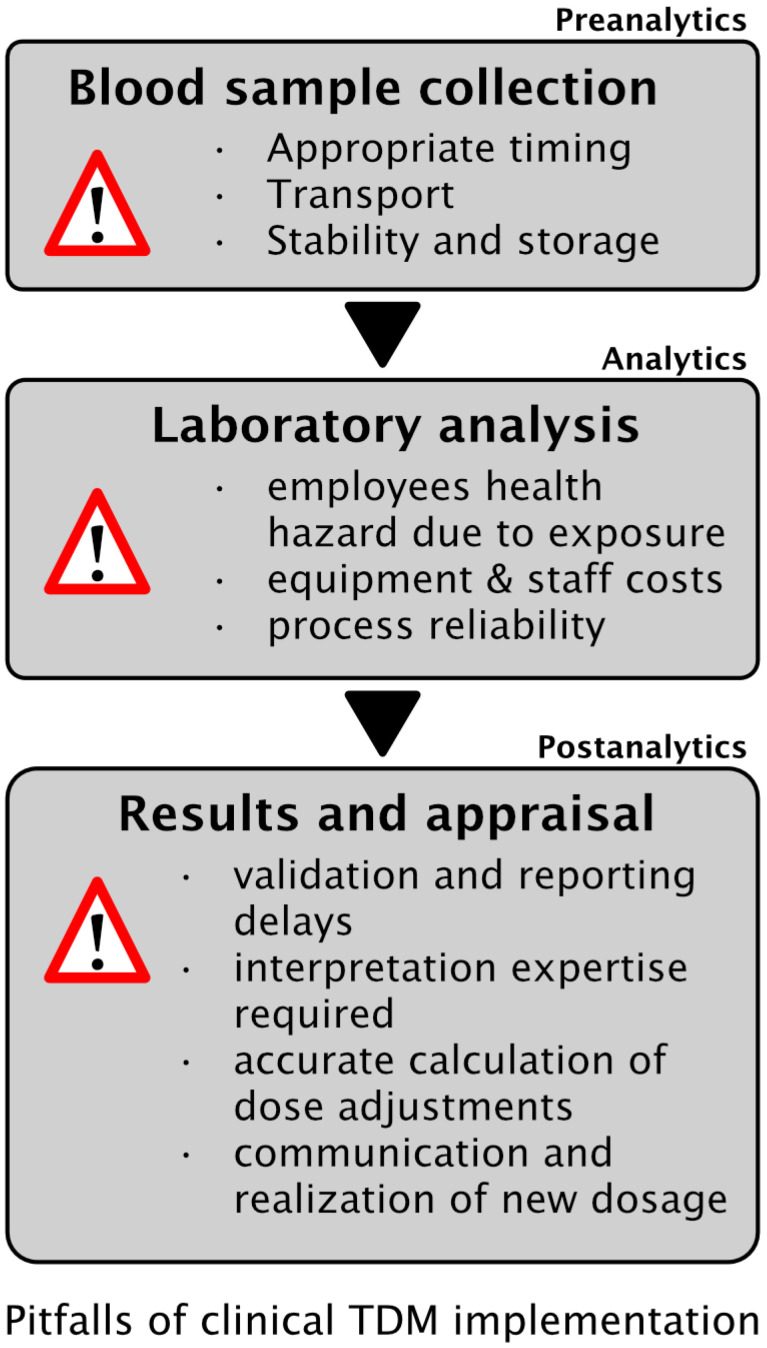
Typical pitfalls and influencing factors of therapeutic drug monitoring from pre- to postanalytical stage.

Another essential element for successful TDM implementation depends on human factors. An optimized and personalized antimicrobial treatment requires increased alertness of the clinicians’ at several steps. The decision to measure piperacillin serum concentration was based on the clinician experience and supported by a regular antimicrobial stewardship team. Nevertheless, 16% of the patients received PIP/TAZ therapy without TDM. Although this might partly be explained by an initiation of therapy limitations, we analyzed the implementation process and illustrated typical pitfalls to improve TDM implementation into clinical routine in the future (Figure 2 and Figure 3).

Another important factor for successful implementation of TDM based therapy is the reasonable adaption of the piperacillin dose to the measured serum concentration. In 65 cases, measurements were repeated before piperacillin doses were adapted. Although this might be attributed to the implementation process, lack of experience of the attending physicians regarding dose adaption might also have been a contributing factor. Thus, one single time-point measurement may differ relevantly from measurements of drug exposure such as the area under the concentration time curve (AUC) [17,42]. To provide more accuracy, the application of pharmacokinetic-pharmacodynamic target-guided dosing based on dosing software might be beneficial [43,44]. For example, the Bayesian forecast software includes individual patient data that were compared to a model-derived population prior probability to estimate individual pharmacokinetic parameters to determine dose adjustment to achieve optimal pharmacokinetic-pharmacodynamic targets [45]. Finally, the application of Bayesian software was reported to achieve improved target attainment compared to fixed dosing strategies. Moreover, a reduction in the required blood samples and increased flexibility around sample times was demonstrated by usage of dosing software [46,47].

## 4. Materials and Methods

### 4.1. Study Design and Patient Population

This report represents a descriptive single center analysis based on data collection for quality control. In January 2020, initiated by our in-house antimicrobial stewardship team, TDM was routinely started for all patients receiving PIP/TAZ treatment by continuous infusion. The implementation process was analyzed in view of practicability, adherence of clinicians to developed standard operating procedures, effect of piperacillin levels on doses adaption, and the patients’ potential benefit. All data were analyzed retrospectively and registered within clinical routine work, using standardized forms to record demographic and clinical characteristics, procedural, and follow-up data. All patients receiving antibiotic therapy with PIP/TAZ at the department were consecutively included in the study. This is a retrospective quality improvement study, therefore, approval was waived by the Institutional Review Board of the University Hospital Wuerzburg, Germany (Number: 20210929 01).

### 4.2. Established TDM Concept for PIP/TAZ

An interdisciplinary team including the in-house antimicrobial stewardship team, pharmacists, laboratory, and intensive care physicians finalized a new standard for TDM of piperacillin. Recommendations and references as to dosing, application items, and measurements of piperacillin levels were based on current data taking the minimal inhibitory concentrations (MIC) of Gram-negative bacteria into account [23,24,48,49].

The indication for commencing antibacterial treatment, its continuation as well as dosing target of piperacillin were continuously reevaluated and adapted by intensive care specialists and supported twice weekly by the antimicrobial stewardship team. The implementation process included a sound instruction to the new local guideline on TDM of piperacillin of the attending physicians and the nurse staff by the hospital pharmacists.

### 4.3. Preparation and Initial Dosing of PIP/TAZ Infusion

The initiation of antimicrobial therapy was at the discretion of the treating physician following a standardized dosing regimen. After reconstitution of 4.5 g PIP/TAZ in 20 mL aqua or sodium chloride, the drug was further diluted by compatible solvents, resulting in a total volume of 50 mL. Although physically stable for several hours, the solution was used immediately after preparation due to hygiene aspects.

The initial dose scheme was intended to exceed blood levels above the MIC rapidly and without dose adaption based on renal function. An initial dose of 4.5 g PIP/TAZ was administered intravenously over 30 min. Simultaneously, a maintaining dose based on renal function was administered. Depending on the glomerular filtration rate (GFR) above or under 20 mL/min, patients received 13.5 g or 9 g PIP/TAZ in 24 h (i.e., 4,5 g piperacillin continuously per 8 or per 12 h change intervals, respectively) [24,32,36].

### 4.4. Drug Level Measurement and Sample Collection

The University Hospital Wuerzburg provides a qualitative in-house-test for piperacillin. Due to the limited stability of blood samples at room temperature, short distances and near-term further processing are essential for valid and reliable test results. Internal validation process showed a maximum stability of 5 h under room temperature and a maximum stability of 8 h between +2 and +8 °C.

The first drug level was measured 6 h after loading dose application at the earliest. Measurements were conducted three times a week (Monday, Wednesday, and Friday), blood samples were collected between 6–8 a.m. After blood collection, the samples were immediately transferred to the central laboratory for further processing and determination of piperacillin levels by isotope dilution HPLC tandem mass spectrometry (HPLC-MS/MS) method. A deadline at 8 am was established due to the fact of pre-analytical quality. The results were available in the afternoon of the same day.

### 4.5. Pharmacokinetic and Pharmacodynamics Targets

Non-species related breakpoints were applied as the lower limit for the therapeutic range. The steady-state concentration for piperacillin was determined, based on the clinically sensible breakpoint against the pathogenic *Pseudomonas* spp. at 16 mg/L and an average protein binding of 30%, by a total minimum drug concentration 100% fT_>MIC_ at 22.5 mg/L [23]. To ensure this target concentration, we implemented a pathogen specific target concentration depending on MIC. During empiric calculated PIP/TAZ therapy, prior to pathogen isolation, piperacillin target concentration was set at 80 mg/L [23,24]. Since drug levels above 100 mg/L increase the risk of side effects, the continuous infusion of PIP/TAZ was reduced, according to the standard operating procedure of the University Hospital Wuerzburg (Figure 2).

### 4.6. Statistical Analysis

All data were transferred from the hospital information system into a pseudonymous database containing baseline patient characteristics (e.g., age, weight, height, co-morbidities, outcome), duration of antimicrobial therapy, laboratory parameters, and further supportive intensive care therapy including dialysis and levels of vasopressor medication.

Implementation data were screened for inadequate deviation from the standard operating procedures and non-adaption of dose regime in dependence of evaluated drug levels. Patient data were analyzed for differences concerning the piperacillin target attainment. All calculations and statistical analysis were performed using IBM SPSS Statistics version 23.0.0.3 software (IBM, Armonk, NY, USA). Data were presented as frequency, distributions, and percentages. All continuous data were presented as mean ± standard deviation (SD). Differences between groups were assessed for statistical significance using the Mann–Whitney U-test and were considered as significant as *p* ≤ 0.05. The coefficient of correlation (ρ) was calculated using the Spearman correlation analysis.

## 5. Conclusions

Optimized dosage of particularly hydrophilic antimicrobial agents in critically ill patients is challenging and a “one fits all” practice might either be dangerous or without avail. Therefore, the application of TDM might improve patient outcomes, avoid drug resistance, and reduce health care costs. However, the success of TDM implementation into clinical practice relies on several factors such as the appropriate timing of sample collection, sample transport to the laboratory, analysis, and processing by HPLC with subsequent reporting. Appropriate dose adaption in accordance to the TDM results requires knowledge and experience from the clinicians. Finally, the application of a dosing adaption software might additionally improve implementation and may alleviate the decision finding process of dose adaption.

## Figures and Tables

**Table 1:** Patient characteristics, clinical, and laboratory parameters of the piperacillin level monitored patients. Data are presented as mean values. *p* ≥ 0.05 was considered as statistically significant. # shows significant difference between the target attainment group (*n* = 194) vs. the sub-therapeutic group (*n* = 6). § shows significant difference between the target attainment group vs. the potentially toxic group exceeding 100 mg/L (*n* = 71).* sign is for multiplication.

TDM-Level	>100 mg/L (*n* = 71)	>22.5–<100 mg/L (*n* = 194)	<22.5 mg/L (*n* = 6)
**Infusion rate (4 g/0.5 g) [mL/h]**	5.88 ± 1.29 (min. 2.1, max. 11.5)	5.68 ± 1.54 (min. 1.1, max. 12.6)	5.5 ± 2.19 (min. 4.2, max. 8.1)
**Age [a]**	66 ± 22 (min. 42, max. 92)	62 ± 14 (min. 21, max 92)	47 ± 10 (min 40, max. 65) #
**Height [cm]**	175 ± 11 (min. 150, max. 195)	174 ± 10 (min. 152, max. 195)	181 ± 9 cm (min. 165, max. 193)
**Weight [kg]**	90 ± 21 (min. 47, max. 140)	91 ± 25 (min. 53, max. 184)	98 ± 19 kg (min. 77, max. 130)
**BMI [kg/m²]**	30 ± 8 (min. 15, max. 62)	30 ± 8 (min. 15, max. 57)	30 ± 6 (min. 24, max. 40)
**Creatinine [mg/dL]**	2.19 ± 0.97 (min. 0.58, max. 4.30) §	1.41 ± 0.84 (min. 0.27, max. 4.26)	0.92 ± 0.56 (min. 12.1, max. 2.01)
**GFR [mL/min/1.73 m²]**	40.9 ± 26.7 (min. 12, max. 150) §	75.4 ± 51.8 (min. 8.6, max. 280)	120.5 ± 70.5 (min. 38, max 252)
**Hemoglobin [g/dL]**	8.6 ± 1.0 (min. 6.9, max. 12.1)	8.9 ± 1.8 (min. 6.3, max. 24.6)	10.2 ± 2.3 (min. 6.9, max. 13.1)
Leukocytes [* 1000/µL]	15.7 ± 8.4 (min. 3.3, max. 42.1)	12.9 ± 6.1 (min. 0.4, max. 32.5)	14.2 ± 6.0 (min. 6.0 max. 23.8)
Thrombocytes [* 1000/µL]	180 ± 99 (min. 42, max. 476)	228 ± 140 (min. 37, max. 968)	246 ± 81 (min. 118, max. 368)
**CRP [mg/dL]**	20.2 ± 11.7 (min. 1.1, max. 52.5) §	16.9 ± 12.0 (min. 0.9, max. 60.5)	5.8 ± 5.1 (min. 1.1, max. 14.1) #
**Procalcitonin [ng/mL]**	18.8 ± 36.6 (min. 0.2, max. 189.5) §	4.8 ± 11.2 (min. 0.1, max. 94.4)	n.d.
**Interleukin-6 [pg/mL]**	453 ± 717 (min. 7, max. 4025)	510 ± 2866 (min. 4, max. 35,500)	49 ± 69 (min. 8, max. 172) #
**Norepinephrine [µg/min]**	8.1 ± 9.3 (min. 0, max. 33)	5.5 ± 9.8 (min. 0, max. 56)	0

## Data Availability

The data presented in this study are available on request from the corresponding author. The data are not publicly available due to the European General Data Protection Regulation (CDPR).
